# Emerging Roles of GluN3B NMDA Receptor Subunit in the Central Nervous System

**DOI:** 10.1007/s12264-025-01523-z

**Published:** 2025-10-26

**Authors:** Yuerou Huang, Junyi Xie, Jiamin Chen, Siman Guo, Yuan Li, Fang Liu

**Affiliations:** 1 Institute of Mental Health and Drug Discovery, Oujiang Laboratory (Zhejiang Lab for Regenerative Medicine, Vision and Brain Health), School of Psychiatry, Wenzhou Medical University, Wenzhou, 325000 China; 2 Brain Health Institute, National Center for Mental Disorders, Shanghai Mental Health Center, Shanghai Jiaotong University School of Medicine, Shanghai, 200030 China; 3 Campbell Family Mental Health Research Institute, Centre for Addiction and Mental Health, Toronto, ON M5T1R8 Canada; 4 Institute of Medical Science, University of Toronto, Toronto, ON Canada; 5 Department of Pharmacology and Toxicology, University of Toronto, Toronto, ON Canada; 6 Department of Psychiatry, University of Toronto, Toronto, ON Canada; 7 Department of Physiology, University of Toronto, Toronto, ON Canada

**Keywords:** NMDA receptors, GluN3B, *Grin3b*, Biophysical properties, Pharmacological characteristics, Psychiatric disorders

## Abstract

GluN3B is the most recently identified subunit of N-methyl-d-aspartate receptors (NMDARs), and gradually it has been found that it may be involved in regulating the development of central nervous system (CNS)-related diseases. Compared with the traditional NMDARs containing only GluN1 and GluN2 subunits, non-classical NMDARs with GluN3 have non-conventional biophysical, trafficking, and signaling properties. As a negative regulatory subunit that diminishes or inhibits classical NMDARs' functions, GluN3B plays important roles in synaptic plasticity and neuronal survival, and may be associated with CNS disorders such as schizophrenia and substance use disorders. However, the number and depth of studies on how GluN3B is involved in the regulation of related diseases are very limited. This review summarizes the expression and physiological characterization of GluN3B-NMDARs and provides an overview of their emerging roles in psychiatric and neuropsychiatric disorders, aiming to provide a basis for understanding disease mechanisms and developing novel therapeutic targets.

## Introduction

**Fig. 1:**
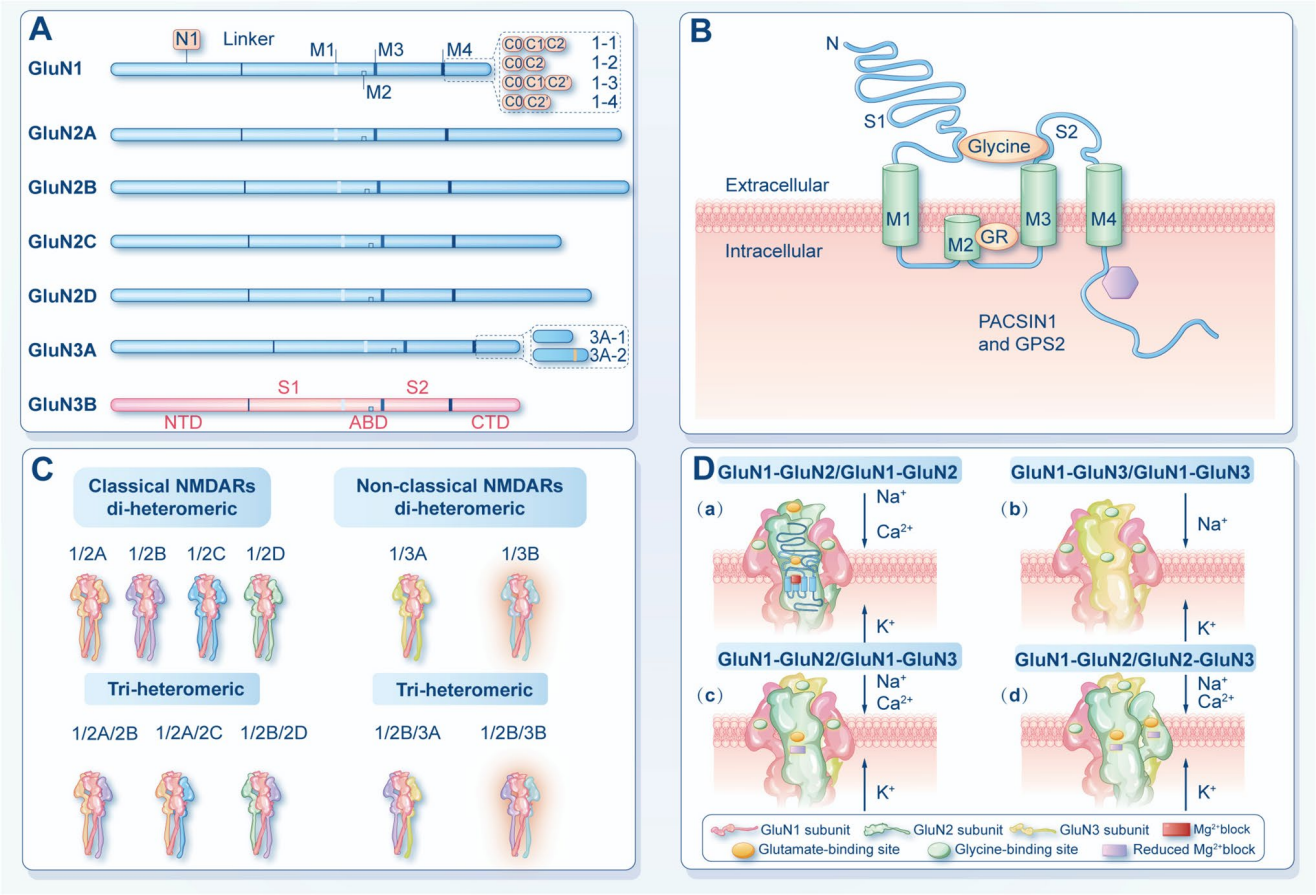
Structure diagram of NMDARs. **A-B** Schematic diagram of the NMDAR subunit domain, GluN3B, contains NTD, ABD, TMD, and CTD. The TMD contains three transmembrane helices (M1, M3, and M4) and a membrane re-entrant loop. **C** The composition of classical and non-classical NMDARs, in which the non-classical NMDARs containing the GluN3 subunit can form di-heteromers and tri-heteromers. **D** Differences in ligand binding sites and ion permeability of different NMDARs.

**Fig. 2:**
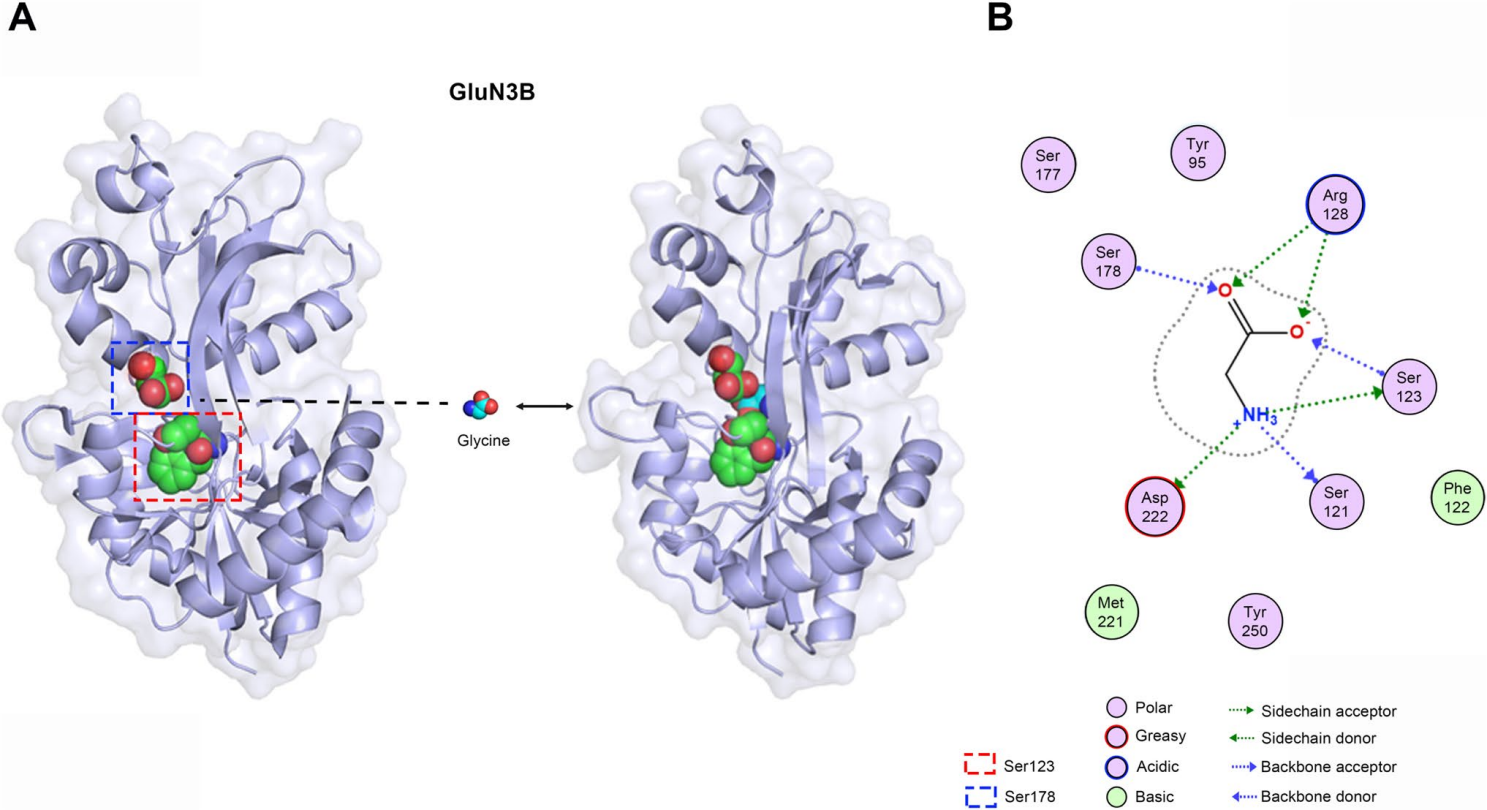
X-ray crystal structure of the ligand binding domain of the GluN3B subunit. **A** The glycine-binding GluN3B LBD. Gromacs2022.3 software was used for molecular dynamics simulation. **B** The interaction diagram between glycine and GluN3B depicts the key residues mediating ligand binding. (ProreinData Bank: 2RCA).

N-methyl-d-aspartate receptors (NMDARs) are major glutamate-gated ion channels, which are essential mediators for brain development and normal functions [1]. To date, seven different subunits of NMDARs have been identified, including the GluN1 subunit, four GluN2 subunits (A-D), and two GluN3 subunits (A-B). The GluN3 subunit, also known as the NR3 subunit, is the latest NMDARs subunit discovered [2]. Between them, GluN3A was first reported in 1995 and was classified as a subunit of NMDARs due to structural similarities with other NMDARs subunits [3, 4]. In the same year, a partial sequence of GluN3B was cloned, and then in 2001, a team of researchers published a relatively complete characterization of the protein, which had the highest similarity to GluN3A, with 51% similarity in mouse [5] and 57% in human [6]. Moreover, the GluN3B subunit shares four distinct structural domains with all other NMDARs subunits: the extracellular N-terminal domain (NTD), which is responsible for subunit assembly and metastable regulation; the extracellular agonist-binding domain (ABD), which binds glycine (or D-serine) in GluN1 and GluN3 subunits and glutamate in GluN2 subunits; the transmembrane domain (TMD), and an intracellular carboxyl-terminal domain (CTD) (Fig. 1A) [7–9]. The GluN3B ligand binding core crystal structures are also common to other members of NMDARs, with the ABD located between the NTD and the TMD, and glycine (or D-serine) binds in the cavity of the ABD (Fig. 1B) [1, 10]. There is one distinct binding site between GluN3B and glycine and molecular dynamics simulations demonstrate that GluN3B engages with glycine to form a stable conformation (Fig. 2A). Among them, GluN3B basic residue Arg128, the acidic residue Asp222 and the polar residues Ser121, Ser123 and Ser178 can form interactions with glycine backbone or sidechain (Fig. 2B) (PDB ID: 2RCA). In *Homo sapiens*, the gene encoding GluN3B is located on chromosome 19p13.3 and contains 2703 bp. Meanwhile, in mice and rats, it is on chromosome 10 and chromosome 7q11, respectively (Data from https://www.ncbi.nlm.nih.gov/). Comparative genomic analyses reveal that the human *GRIN3B* gene is longer than its murine counterpart, primarily due to extended intronic regions [11]. Despite this structural divergence, the protein-coding sequence of GluN3B exhibits moderate evolutionary conservation, with 81.6% nucleotide identity between humans and mice. However, this conservation level is lower than that observed for the other six NMDA receptor subunits [11]. Moreover, compared to other NMDARs subunits, GluN3 (including GluN3A and GluN3B) has the most similar modular architecture to GluN1 in that they both bind glycine or D-serine [12].

Since the subunits of NMDARs can bind to each other into different functional receptors and change dynamically with development, NMDARs have a profound effect on neuroplasticity in the developing and adult brain [1]. The precise assembly rules of NMDARs remain controversial, and it is frequently suggested that functional NMDARs can be assembled with two GluN1 subunits, two GluN2 subunits, or the relatively rare GluN3 subunits [2, 13]. The NMDARs containing two GluN1 subunits and two GluN2 subunits are referred to as classical or conventional NMDARs. In contrast, the NMDARs containing two GluN1 subunits and two GluN3 subunits (Di heteromeric GluN1-GluN3 complexes) and tri-heteromeric GluN1-GluN2-GluN3 complexes containing two GluN1 subunits, one GluN2 subunit, and one GluN3 subunit or one GluN1 subunit, two GluN2 subunits, and one GluN3 subunit are referred to as non-classical NMDARs (Fig. 1C) [14, 15]. Currently reported GluN1-GluN2-GluN3 tri-heteromeric NMDARs typically have two GluN1 subunits [13, 14]; however, some studies have also identified forms containing two GluN2 subunits [2, 16]. The functional significance of NMDA receptors arises from their defining characteristics: their Mg^2^⁺ block (requiring prior depolarization for relief), their dependence on a coagonist, and their high Ca^2^⁺ permeability [17]. Compared with classical NMDARs, NMDARs containing GluN3A and GluN3B have reduced Ca^2+^ influx and are less sensitive to voltage-dependent block by Mg^2+^ (Fig. 1D) [14, 18]. Due to these unique properties, GluN3A and GluN3B are considered dominant-negative subunits that antagonize the function of classical NMDARs [14]. In heterologous expression systems, GluN3 can form excitatory glycine-gated receptors with GluN1, and these unusual glycine receptors may mediate the atypical functional traits observed in NMDARs containing GluN3 [17]. For these two nonclassical NMDARs, reviews regarding GluN3A-containing NMDARs have been elaborated elsewhere [14, 19]. Here, we will provide an overview of the physiological properties of GluN3B. Furthermore, we will review the current evidence linking GluN3B dysfunction to a variety of psychiatric disorders and discuss the possible underlying mechanisms. We additionally consider how GluN3B may more broadly affect the normal function of the body besides the brain from the limited information obtained from the biological databases.

## Expression and Physiological Features of GluN3B

**Fig. 3:**
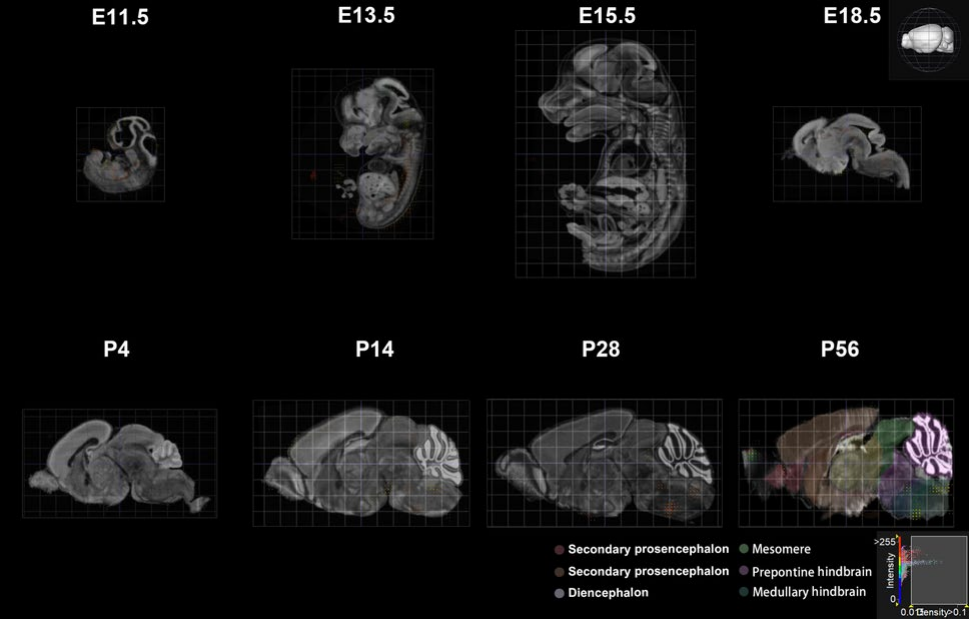
The expression pattern of *Grin3b* during development. *Grin3b* is expressed during the embryonic stage, reaches its lowest level at P0-P1, begins to increase from P7, and persists into adulthood. As mice continue to develop, *Grin3b* is primarily expressed in the medullary hindbrain, mesomere, and pallium. Each dot represents the expression of *Grin3b*, with the color indicating the level of expression. E, embryonic day; P, postnatal day.

In the central nervous system (CNS), GluN3B shows spatiotemporal distribution differences during the developmental and adult periods. During development, *Grin3b* can be detected as early as the embryonic stage (Fig. 3, created from the Allen Brain Atlas). However, some studies have reported no significant *Grin3b* mRNA signals in the mouse brain and spinal cord from E15 to P7 [20]. Therefore, further research is needed to clarify the expression profile of *Grin3b* during the early developmental period. In contrast, the postnatal expression pattern of GluN3B is relatively well-established: it is lowest during P0-P1, increases rapidly between P7 and P14, peaks at approximately six weeks of age in rodents, and persists into adulthood [20–22]. As mice continue to develop, *Grin3b* is primarily expressed in the medullary hindbrain, mesomere, and pallium, with the highest expression in the pallium during adulthood (Fig. 3, created from the Allen Brain Atlas). This differs in the temporal distribution of GluN3A, in which the expression level peaks one week after birth and then gradually declines with age [15, 23]. These results indicate that GluN3B and GluN3A have completely different protein expression patterns, although they are both GluN3 subunits.

**Fig. 4:**
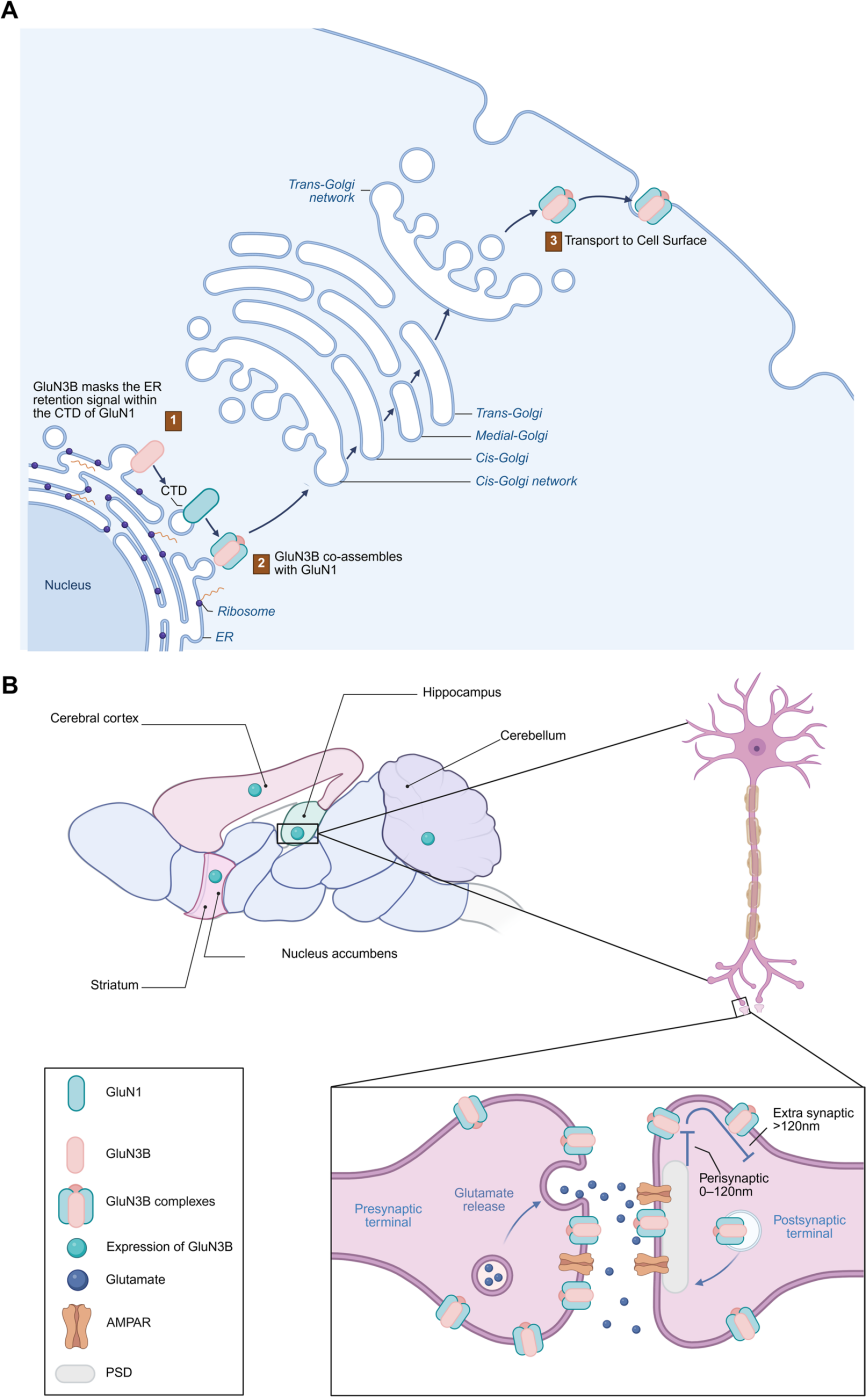
The expression and distribution of GluN3B in the CNS and subcellular structures. **A** GluN3B requires co-assembly with GluN1 for ER export and surface localization. **B** GluN3B is widely distributed throughout the hippocampus, cortex, nucleus accumbens, striatum, and cerebellum. Regarding subcellular localization, GluN3B is present in both presynaptic and postsynaptic areas. Immunoelectron microscopy data further support that GluN3B is more enriched in presynaptic terminals (e.g., hippocampal mossy fibers) and localized preferentially at the synaptic perimeter (60–120 nm from PSD center). (Created in https://BioRender.com).

As for the specific expression site of GluN3B, it was once thought to be specifically expressed only in motor neurons on the brainstem and spinal cord [5, 21, 24], but in recent years, studies have found a more widespread distribution of GluN3B in the CNS, including the hippocampus, striatum, cerebral cortex, nucleus accumbens and cerebellum in mammals (Fig. 4B) [11, 25]. Researchers claimed that the expression pattern of GluN3B may be as ubiquitous as that of GluN1 [20, 25]. This can be further demonstrated by the later studies that GluN3B was found to be expressed in pyramidal neurons of the hippocampus and cortex, in parvalbumin and somatostatin-expressing interneurons of the hippocampus, striatum, cerebellum, and nucleus accumbens, as well as in cholinergic interneurons within the striatum [25]. These all indicate that GluN3B-containing NMDARs may be abundantly expressed within the CNS, suggesting a versatile role of GluN3B in regulating the normal and pathological function of the brain.

Regarding the subcellular distribution of GluN3B, much less information is available currently. GluN1 cannot be transported to the cell surface alone, and the endoplasmic reticulum (ER) retention signal of the CTD of the GluN1 subunit needs to be masked by GluN2 or other proteins so that the cell surface transport of the NMDARs complex can be precisely controlled. Whereas GluN3B needs to be separated from the endoplasmic reticulum and transported to the cell surface must also bind GluN1 (Fig. 4A), although the CTD of GluN3B is not responsible for co-assembly with GluN1, its region between amino acids 952-985 may mask the endoplasmic reticulum retention signal of GluN1 [26, 27]. Thus, the two bindings are complementary, and together they facilitate membrane localization and cell surface translocation of GluN3B.

For the ultrastructural localization of GluN3B, a study of immunolabeling and biochemical isolation of hippocampal neurons in rats showed that GluN3B is mainly located at the synaptic site, and its expression increased as neurons mature [22]. Another study also supports the expression of GluN3B at the synaptic site [25]. For the postsynaptic membrane, the post-synaptic density protein of 95 kDa (PSD-95) is a critical synaptic protein for functional NMDARs localization at synapses, in which the PSD-95/discs large/ZO-1 homologous (PDZ) domain connects NMDARs to PSD, and these proteins share a key role in regulating the synaptic localization and plasticity of NMDARs [28, 29]. To further investigate the intrasynaptic distribution of GluN3B, a biochemical fractionation study showed that compared to traditional NMDARs aggregations [24], both GluN3A and GluN3B are expressed at a lower level in the core of PSD and that the density of GluN3B particles increases towards the edge of PSD [5, 14]. However, no PSD-95 binding motif has been detected at the C-terminal of the GluN3B protein sequence [22], which means GluN3B alone cannot be co-expressed with PSD-95 (Fig. 4B). Similarly, GluN3A does not contain a PDZ domain that binds to PSD-95 either [19, 22]. This all suggests that GluN3B is a peripheral binding protein in the synaptic region.

In addition, another study claimed that GluN3B is present not only in the periphery of the postsynaptic membrane PSD but also in the presynaptic membrane with certain synapses (Fig. 4B) [30]. Considering the widespread expression of GluN3B in the hippocampus, immunocytochemistry was used to localize GluN3B in rat hippocampal mossy fibers and recurrent associational/commissural synapses (rA/C synapses). Studies have observed that the distribution density of gold particles of GluN3B on the mossy fiber synapses is relatively high and inclines toward the presynaptic direction [30]. Further research found GluN3B in the vesicular structure of the cytoplasm of the mossy fiber terminals, moreover, GluN3B has a strong presynaptic localization compared to most NMDARs that are located mainly in the postsynaptic membrane [30] (Fig. 4B), a phenomenon that favors its localization in the presynaptic region and may reflect the transport of the receptor in and out of the plasma membrane. It also suggests that the presynaptic mossy fiber membranes are more likely to be composed of GluN1 and GluN3B subunits.

## Receptor Assembly and Biophysical Properties

Most natural NMDARs are composed of a heterotetramer assembly of two GluN1 subunits and two GluN2 subunits. In heterologous cells, GluN3B can associate with various NMDARs subunits, but it must co-assemble with GluN1 to form a receptor complex that can be released from the endoplasmic reticulum to the cell membrane [31]. The known receptor complexes containing GluN3B are mainly divided into two types: the di-heteromeric GluN1-GluN3B receptor and the tri-heteromeric GluN1-GluN2-GluN3B receptor.

### The Di-heteromeric GluN1-GluN3B Receptor

The di-heteromeric GluN1-GluN3B assembly exhibits a unique property of non-responsiveness to NMDA or glutamate, necessitating the presence of glycine (or D-serine) for its activation. Consequently, this complex does not adhere to the conventional classification of NMDARs, instead constituting a novel class of glycine-gated excitatory receptors. This observation has sparked considerable intellectual curiosity and academic debate among researchers [32, 33]. However, to date, the majority of studies on these receptors have been confined to recombinant systems, with scarce evidence supporting their in vivo existence. Moreover, the atypical GluN1-GluN3B receptors generate feeble and unstable glycine currents, posing a challenge for accurate quantification and adding complexity to research endeavors [34]. Although GluN1/GluN3B receptors can mediate glycine-gated excitatory currents, their sensitivity to glycine is relatively low. According to literature reports, conventional GluN1/GluN2 receptors exhibit a glycine EC50 value of 0.1–2 µmol/L [35], while GluN1/GluN3A has an EC50 value of 7.1 ± 0.4 µmol/L [36]. However, the exact EC50 value for GluN1/GluN3B remains unreported. Some studies predict an EC50 of approximately 5 µmol/L [37]. Additionally, in cells transfected with NR1/NR3A/NR3B subunits, the calculated EC50 values for glycine-activated peak and steady-state currents were 44.3 and 14.0 µmol/L, respectively [38]. The high EC50 value of GluN3B is attributed to mutations in key residues of its glycine-binding pocket, which reduce binding affinity [10]. This difference suggests that under resting synaptic glycine levels (1–3 µmol/L) [39], GluN1/GluN3B remains largely inactive. This property enables its preferential activation in extrasynaptic high-glycine environments, such as during pathological conditions with elevated glycine concentrations.

From a pharmacological perspective, GluN1-GluN3 receptors confront the scarcity of potent and selective antagonists. Currently, available potentiators exhibit limited efficacy in altering the transient nature of GluN1-GluN3 responses and often lead to complex biphasic reactions [40]. Notably, while glycine is generally recognized as an inhibitory neurotransmitter in the spinal cord and brainstem [32], it assumes dual roles as both an agonist and a functional antagonist in GluN1-GluN3B receptors. This unique mechanism, wherein the opposing effects triggered by glycine binding to distinct subunits lead to rapid receptor desensitization akin to a self-inhibitory state, imparting unusual concentration-dependent and temporal characteristics to the current [41].

Specifically, this mechanism underpins the emergence of bell-shaped dose-response curves and distinct desensitization and resensitization phases, also known as "tail currents," observed during agonist application and washout, respectively. Nevertheless, how this intricate dynamic behavior translates into specific physiological effects remains an uncharted territory [42].

Intriguingly, the activation paradigm of GluN1-GluN3 receptors diverges significantly from that of conventional GluN1-GluN2 NMDARs. In the latter, the occupancy of multiple agonist binding sites among the four is necessary for receptor activation, whereas in GluN1-GluN3 receptors, mere engagement of the two constitutive GluN3 subunit binding sites suffices to trigger activation [36]. This atypical agonist dependency underscores novel structural and mechanistic foundations, offering fresh perspectives while posing additional enigmas.

Given the distinctive activation mechanism and its implications for understanding GluN1-GluN3 receptor functions in health and disease, a crucial step towards unlocking these secrets is the development of high-affinity, specific antibodies that can selectively target and study these receptors. Nevertheless, the development and validation of these specific antibodies are still in their infancy, necessitating further endeavors to overcome technical hurdles and propel research advancements in this field.

### The Tri-heteromeric GluN1-GluN2-GluN3B Receptor

Despite the incomplete understanding of the precise stoichiometry of the tri-heteromeric GluN1-GluN2-GluN3B complex, most current researches suggest that it is assembled as a tetramer consisting of two GluN1 subunits, one GluN3B subunit, and one GluN2 subunit [1, 2]. Electrophysiological studies of pure populations of GluN1-GluN2-GluN3B are challenging and complex due to the unavoidable co-expression of di-heteromeric receptors when tri-heteromeric receptors are expressed. Moreover, studies have shown that di-heteromers and tri-heteromers coexist within single cells, and even at individual synapses [1]. To address this challenge, new methods have been developed that use ectopic retention signals to allow the heterologous expression of the necessary tri-heteromeric receptors [43, 44]. If these methods are widely adopted, it would be a significant advancement.

The tri-heteromeric receptors respond to NMDA and glutamate, thus bona fide NMDARs. However, they exhibit non-classical biophysical characteristics in both heterologous cells and neurons. For example, when the GluN3B subunit is co-expressed with GluN1-1a and GluN2A, it increases sensitivity to glycine [37]. Additionally, studies have shown that when the GluN3B is co-expressed with GluN1 and GluN2 in heterologous HEK293 cells, the GluN3B subunit reduces the amplitude of NMDA-induced currents and lowers the Ca^2+^ permeability of the NMDARs complex, producing smaller whole-cell currents elicited by glutamate than classical NMDARs [5]. Similar phenomena have been observed when GluN3B co-assembles with endogenous GluN1 and GluN2A in hippocampal neurons [24]. The Ca^2+^ influx through NMDARs is believed to initiate processes leading to synaptic plasticity, and if the receptors are overloaded, it can lead to certain forms of neuronal death. Therefore, NMDARs expressing GluN3B may play an important regulatory role in synaptic plasticity and neuronal death by altering their Ca^2+^ permeability. Furthermore, receptors containing GluN3 subunits are also relatively insensitive to the block by magnesium at hyperpolarized potentials. Further studies have found that when cRNA of rat NMDARs subunits is injected into Xenopus oocytes, receptors containing GluN3B subunits are less sensitive to magnesium than those containing GluN3A subunits [45]. Recently, the structural basis of the magnesium block mechanism in NMDA receptors has been elucidated by Huang *et al*. [46]. Mg^2^⁺ primarily exerts its effects through voltage-dependent blockage of the NMDARs channel, a mechanism largely attributed to the “glutamine-arginine-asparagine (QRN)” site [47]. The key asparagine residues within the QRN sites of GluN1 and GluN2 subunits can form coordination bonds with Mg^2^⁺, thereby facilitating Mg^2^⁺ blockage. However, the QRN sites in GluN3 subunits lack asparagine residues (for instance, GluN3B may have arginine or glutamate at the corresponding positions), and thus cannot form similar coordination bonds with Mg^2^⁺ [46]. This structural difference renders the GluN3B subunit insensitive to Mg^2^⁺, meaning that the receptor channels formed by GluN3B remain open even in the presence of high concentrations of Mg^2^⁺.

In summary, owing to their distinctive properties, GluN3B has been proposed as a predominant negative subunit that antagonizes the functionality of classical NMDARs. This implies that when GluN3B coexists and assembles with the canonical NMDARs subunits, such as GluN1 and GluN2, they may diminish or inhibit the normal functions of these receptors. This antagonistic effect is potentially mediated through alterations in channel ion permeability, modulation of receptor activation thresholds, or alterations in receptor distribution within the cell, among other mechanisms.

## Pharmacological Characteristics of the GluN3B Subunit

### Development of Antagonists

Traditionally, Mg^2+^, memantine, and MK-801 are well-recognized NMDARs antagonists [48]. However, these drugs exhibit poor antagonism against NMDARs containing GluN3 subunits, even at concentrations that strongly inhibit classical NMDARs [37, 38]. Given the exceedingly high sequence homology between GluN3A and GluN3B, the endeavor to devise specific activators or inhibitors targeting GluN3B poses formidable challenges. Nevertheless, research has revealed that unclassical NMDARs containing the GluN3B subunit display higher resistance to these antagonists than those containing the GluN3A subunit, potentially due to several differences between the GluN3A and GluN3B sequences in the M2-M3 region that define the narrow part and vestibule of the pore [45, 49]. Currently, there are relatively few allosteric modulators targeting GluN3B. However, some studies have identified potential regulatory molecules, such as the non-specific antagonists EU1180-438, [2-hydroxy-5-((4-(pyridin-3-yl)thiazol-2-yl)amino]benzoic acid (TK13) and 4-(2,4-dichlorobenzoyl)-1H-pyrrole-2-carboxylic acid (TK30) targeting GluN3 [50, 51].

To identify these influential regions, researchers have developed a novel method by mutating the orthosteric ligand-binding pocket in the GluN1 subunit, specifically designed to study the pharmacological properties of the GluN3 subunit in recombinant GluN1-N3 dimeric receptors. The F484A and T518L mutations in GluN1 significantly enhance the peak and steady-state currents while suppressing the desensitization of the GluN1-GluN3B receptors [50]. These mutations prevent the receptor from rapidly entering a desensitized state at high glycine concentrations [52], thereby facilitating the study of the pharmacological properties of the GluN3 subunit. Using this method, they successfully identified a novel competitive antagonist, 6-hydroxy-[1,2,5]oxadiazolo[3,4-b]pyrazin-5(4H)-one (TK80). The antagonist exhibits high selectivity for the GluN3B subunit, as evidenced by its IC50 value of 79 µmol/L for the GluN1^(F484A/T518L)^/N3B receptor. In contrast, its IC50 values for the GluN1^(F484A/T518L)^/N3A receptor and the GluN1/GluN2 receptors are all greater than 300 µmol/L, indicating no significant inhibitory effects on these receptors [41].

Additionally, a high-throughput calcium ion assay based on cellular systems has been employed to screen for novel GluN3-NMDARs inhibitors. Among 2560 compounds, WZB117 is identified as a relatively selective allosteric inhibitor of GluN1/GluN3 receptors, with an IC_50_ value of 1.15 ± 0.34 µmol/L for GluN3A in the presence of 100 µmol/L glycine at pH7.3 [53]. Due to the failure to record currents of WT GluN1/GluN3B receptors, the GluN1-4a^F484A/T518L^/GluN3B mutants were used to investigate the effects of WZB117. It was found that at a concentration of 30 µmol/L, WZB117 significantly inhibited the currents mediated by the GluN1-4a^F484A/T518L^/GluN3B receptor [51, 53].

Although these technologies are still nascent, developing GluN3B-specific inhibitors/agonists holds great significance. They propel novel drug development strategies and deepen our understanding of the intricate NMDARs mechanisms in the nervous system.

### Studies on Functional Characteristics

Research combining electrophysiology and rapid solution exchange techniques has found that N-glycosylation sites alter the functional properties of GluN1-GluN3B receptors but not GluN1-GluN3A receptors. Notably, the Aleuria Aurantia Lectin (AAL) has a significant impact on GluN1-GluN3B receptors, an effect partially mediated by the N-glycosylation site at N465 on the GluN3B subunit. Furthermore, 7-MEOTA, a compound with potential therapeutic value for Alzheimer’s disease (AD) treatment [54], exhibits higher potency on GluN1-^F484A^-GluN3B receptors than on GluN1-^F484A^-GluN3A receptors [55].

## GluN3B and Central Nervous System Disorders

Due to their important roles in synaptic plasticity and neurodevelopment, NMDARs have been found to be associated with a variety of central nervous system disorders. However, currently, research on GluN3B or GluN3B-containing NMDARs is relatively limited, and most of the articles are about their putative roles in schizophrenia, substance use disorders, and other psychiatric disorders. Herein, we summarize recent findings that shed light on how GluN3B dysfunction might participate in the pathogenesis of these diseases.

### Schizophrenia (SCZ)

SCZ is one of the most common psychiatric disorders, and mounting evidence suggests that the abnormalities of glutamatergic transmission underlie the phenotypes of SCZ. Numerous studies demonstrated that decreased NMDARs expression and NMDARs antagonists are sufficient to cause SCZ-like phenotype in both humans [56, 57] and rodents [58, 59]. For example, the NMDAR antagonist ketamine can produce behaviors similar to symptoms of SCZ (e.g., behavioral and cognitive deficits) in healthy human subjects [60]. As well as impaired GluN2A transport into neuronal dendrites mediated by KIF3 can cause schizophrenia-like phenotypes in mice [61]. Moreover, mice with low GluN1 expression exhibit behaviors associated with schizophrenic movement, including increased motor activity and stereotypy, and deficits in social and sexual interactions [62].

However, almost all the researches are focused on the pathological roles of the dysfunction of subunits of classical NMDARs [63–65], with few studies suggesting the involvement of dysregulated GluN3B in SCZ. For example, in 2011, J Tarabeux *et al*. [66] showed that approximately 10% of the SCZ patients were homozygous for a truncating mutation of the *GRIN3B* gene. Although there is a relatively high rate of mutation for SCZ, this is almost the same proportion as described in some normal healthy human populations [67]. Therefore, some researchers claimed that the role of the *GRIN3B* gene in SCZ may be unlikely. However, to explore the interactions of anti-psychotic drugs and their target genes in SCZ, a systematic investigation found that genes including *GRIN2A*, *GRIN2C*, and *GRIN3B* involved in neuroactive ligand-receptor pathways, glutamate metabolism, and glycine metabolism could be significantly affected by the treatment of anti-psychotic drugs [68]. This suggests the possibility of GluN3B dysfunction in the pathology of SCZ.

Further, genetic screening of SCZ patients subsequently identified a shift mutation (rs10666583) in *GRIN3B* associated with an increased risk of SCZ, as the mutation rate was significantly increased in SCZ patients compared to healthy controls and could lead to genetic susceptibility to SCZ [69–71]. In addition, the mutation in *GRIN3B* (rs2240158) also showed a significant correlation with SCZ-related symptomatic auditory mismatch negativity (MMN) [72], but this needs to be further validated due to the limited sample size. Altogether, the above studies fully illustrate the possibility of malfunction of GluN3B in the pathology of SCZ.

### Substance Use Disorders

Except for SCZ, GluN3B is also closely associated with another CNS disorder, substance use disorder. It is well known that long-term use of opioids such as cocaine can lead to dependence and a "craving" for the drug after withdrawal [73], and in fact, it has been shown that GluN3B expression varies in these addicted and withdrawn groups [74, 75]. After binge cocaine self-administration in rats, the expression of GluN3B increased in a time-dependent manner only in the striatum. Although it decreased after 2 weeks of withdrawal, it was still higher than that in the control group, suggesting that the effects of cocaine abuse on GluN3B may be long-lasting and brain region-specific [74]. Consistent with this hypothesis, GluN3B expression in human peripheral blood lymphocytes was also significantly upregulated in opioid addicts and abstainers, which may be a long-term consequence of opioid abuse, and suggests that GluN3B in peripheral blood lymphocytes may serve as a marker for opioid addiction [75]. Furthermore, the genetic variant rs2240158 of *GRIN3B* was also shown to be associated with a higher risk of heroin addiction, but not with AD [76, 77]. However, this phenomenon seems to exist only in drug addiction, as there are no significant changes in GluN3B expression in human blood lymphocytes in people with behavioral addictions like computer games [78].

The mechanistic links between GluN3B upregulation and substance use disorders are intriguing and warrant further exploration. Following substance use disorders, upregulation of GluN3B in brain regions (e.g., the striatum) and peripheral blood lymphocytes has been observed. One possible mechanism involves the impact of GluN3B on synaptic plasticity, which is a critical process in the development and maintenance of addiction [79]. After a single cocaine exposure, the amplitude of NMDAR-mediated excitatory postsynaptic currents (EPSCs) onto dopamine (DA) neurons in the ventral tegmental area (VTA) is reduced [80]. This plasticity change is attributed to the lower calcium permeability of GluN3A-containing NMDARs, which reduces the influx of Ca^2+^ into the postsynaptic compartment [81]. GluN3B also possesses this Ca^2+^ property, suggesting that GluN3B may similarly affect synaptic plasticity by reducing Ca^2+^ influx, thereby participating in substance use disorders. However, the precise mechanisms by which GluN3B influences synaptic plasticity and addiction-related behaviors remain to be further investigated.

### Other CNS Disorders

In addition to the above-mentioned research about GluN3B in these pathological conditions, currently, there is limited knowledge of the roles of GluN3B in other CNS disorders, and only isolated reports exist.

In depression-related studies, rats subjected to chronically unpredictable mild stress (CUMS), a classical animal model for studying depression, show a reduced expression of GluN3B in the medial prefrontal cortex (mPFC), suggesting a critical role of GluN3B in mPFC in the onset and progression of depression [82]. Meanwhile, a study found that depressive-like behaviors had appeared in an early AD mouse model with genetic dysregulation of *Grin3b* [83]. Furthermore, at the cognitive level, a study on the correlation between the genetic variations and expression of genes involved in synaptic plasticity and cognitive function concluded that the higher expression of plasma calcium/calmodulin-dependent protein kinase IIα (CaMK2A) in students with better cognitive functioning may be mediated by altering the expression of NMDARs subunits, including *Grin3b* [84]. The levels of GluN3B were also significantly higher in the cerebrospinal fluid of some epileptic patients with significantly impaired cognitive function than in the control group [85]. Interestingly, in a pathological model mouse of Mn-induced learning and memory dysfunction, it has been demonstrated that reduced mRNA stability of *Grin1* and *Grin3b* mediated through down-regulated fat mass and obesity-associated protein (FTO) and leading to impairment of synaptic plasticity and cognitive deficits in hippocampal neurons, provides a potential approach to psychiatric disorders triggered by environmental exposures [86].

In addition, some other studies have shown the presence of shifted code mutations in *GRIN3B* in a very small population (1/68, 1/135) of anorexia nervosa (AN) patients [87, 88]. Apart from these studies, the expression quantitative trait loci (eQTL) variant of *GRIN3B* rs10401454 has been shown to be associated with post-traumatic stress disorder (PTSD) and predicts recovery of PTSD patients after trauma, and thus can be used as a potential biomarker of PTSD [89].

In another animal model, researchers exposed experimental animals to predator odors (e.g., 2,4,5-tri-methyl-thiazoline (TMT)) as a stressor, inducing fear-like behaviors and stress response (elevated corticosterone). This mimics neurobiological changes related to stress, offering a model for studying neuropsychiatric disorders [90–93]. Studies have shown that following TMT exposure, male rats exhibit significant upregulation of mRNA *Grin3b* in both the insular cortex and bed nucleus of the stria terminalis (BNST). Pre-treatment with a mGlu3 receptor negative allosteric modulator (VU6010572, NAM) specifically blocks this upregulation in the insular cortex but not in BNST, indicating mGlu3 as a crucial regulatory point in the former and independent mechanisms in the latter [94–96]. Considering the insular cortex's involvement in negative affect, anxiety, fear memories, and threat conditioning, along with its reciprocal projections with BNST, both regions contribute to emotional behavior measurements in rodents [97, 98]. This finding uncovers the specific regulation of *Grin3b* expression by TMT exposure. It offers a novel perspective on understanding stress response and anxiolytic mechanisms.

It is important to note that there are also a number of psychiatric disorders that *Grin3b* may be associated with, but this has not yet been elucidated. For example, autism spectrum disorders (ASD) may be due to abnormal neuronal transmission, and one study reported that truncating mutations in *GRIN3B* were detected in both ASD and healthy control patients [66], but in another study, it was detected that NRXN1α-deficient ASD patients had down-regulated *GRIN3B* and dysregulated glutamatergic signaling [99]. Meanwhile, in monoamine oxidase A knockout mice, which have been shown to produce autistic-like behaviors, *Grin3b* was significantly down-regulated in postnatal P1 of the mice [100], suggesting that *Grin3b* may affect brain development and function by regulating protein expression and influencing excitatory neuron firing. However, the sample size of this article mentioned above (Avazzadeh *et al*., 2021) was very limited, with only three patients with ASD participating in the study, so the possibility that *Grin3b* is involved in regulating ASD needs to be further determined (Table 1).

**Table 1:** Glycine EC50 Values Across NMDAR Subtypes.

Receptor Type	Glycine EC50 (μmol/L)	Activation Mechanism	Refs
GluN1/GluN2A	1.31	Co-agonist (requires glutamate)	[35]
GluN1/GluN2B	0.72	Co-agonist (requires glutamate)	[35]
GluN1/GluN2C	0.34	Co-agonist (requires glutamate)	[35]
GluN1/GluN2D	0.13	Co-agonist (requires glutamate)	[35]
GluN1/GluN3A	7.1±0.4	Glycine-only	[36]
GluN1/GluN3B	5(predicted)	Glycine-only	[37]

This table provides a comparative summary of glycine sensitivity across NMDAR subtypes, demonstrating that GluN3-containing receptors possess significantly reduced glycine sensitivity relative to traditional NMDARs.

Although there are not many reports related to the biological and pathological function of GluN3B, it is concluded from the published findings that GluN3B may be involved in SCZ, addiction, emotional and cognitive disorders, and other related diseases. This involvement is mainly associated with *Grin3b* mutations and changes in GluN3B protein expression (Table 2). Further preclinical studies need to be conducted to illustrate the causal link between GluN3B dysfunction and these diseases and the underlying mechanisms, which can provide new insights into the function of the *Grin3b* gene and novel explanations for the pathology of related diseases.

**Table 2:** GluN3B as a potential biomarker in neuropsychiatric diseases.

Disease	Sample origin	Potential mechanism	Expression change	Refs
SCZ	Human blood	*GRIN3B* mutation(rs10666583)	(-)	[69–71]
	Human blood	The *GRIN3B* mutation is significantly associated with MMN	(-)	[72]
Substance use disorders	Rat brain	(-)	↑	[74]
	Human blood	(-)	↑	[75]
	Human blood	*GRIN3B* mutation(rs2240158)	(-)	[76]
Depression	Rat brain	(-)	↓	[82]
Cognitive function	Human blood	*GRIN3B* mutation increases CaMK2A levels	(-)	[84]
	Mouse brain	FTO decline inhibits *Grin3b* through the m^6^A modification pathway	(-)	[86]
Epilepsy	Human CSF	GluN3B expression changes	↑	[85]
AN	Human blood	*GRIN3B* mutation	(-)	[87]
	Human blood	*GRIN3B* mutation	(-)	[88]
PTSD	Human blood	*GRIN3B* mutation (rs10401454)	(-)	[89]

(-): Mechanism or GluN3B expression unknown; Arrows indicate alterations of detectable GluN3B in the pathological sample: “↑” is an increase and “↓” is a decrease. SCZ: Schizophrenia; CSF: Cerebrospinal fluid; AN: Anorexia nervosa; PTSD: Post-traumatic stress disorder; m^6^A: N6-methyladenosine.

### Neuroprotection of GluN3B

Given that GluN3B may be involved in regulating the development of certain central nervous system diseases, its neuroprotective effects can be exerted by modulating GluN3B expression. Studies have shown that in rat models of cerebral ischemia-reperfusion (I/R) injury, GluN3B expression in the cortical penumbra region is significantly upregulated, and this upregulation is further enhanced by progesterone treatment, highlighting the potential importance of GluN3B in neuroprotection [101]. In a separate study, GluN3B protein expression was markedly downregulated in the ischemic cortex and hippocampus of rats [102]. However, pretreatment with clonidine, a classical α2-adrenergic receptor (α2-AR) specific agonist clinically used for hypertension [103], significantly upregulated GluN3B protein expression and effectively alleviated brain damage in rat models of cerebral I/R injury. Notably, when co-administered with yohimbine, a selective presynaptic α2-AR antagonist [104], the neuroprotective effects induced by clonidine and its upregulation of GluN3B were reversed. These findings not only reinforce the crucial role of GluN3B subunits in neuroprotection but also suggest that clonidine may exert its neuroprotective effects by upregulating GluN3B through α2-AR activation. Nevertheless, the exact mechanism underlying this effect requires further in-depth investigation to fully elucidate [105, 106].

In conclusion, the GluN3B subunit, owing to its distinctive pharmacological traits, stands out as a promising therapeutic target for addressing neuropathological conditions such as excitotoxicity and cognitive decline [17, 107]. By embarking on the development of highly selective GluN3B antagonists and conducting rigorous research to unravel its intricate functional roles within the nervous system, we aim to not only deepen our comprehension of GluN3B's mechanisms but also pave the way for innovative drug discoveries that can effectively combat neurological diseases.

### Phenotypic Changes in *Grin3b* Knockout Mice

To more intuitively observe the impact of *Grin3b* knockout on related physiological functions, a study constructed *Grin3b* knockout mice and conducted comprehensive health status and behavioral tests on them [26]. In this study, it was found that the fall latency of *Grin3b* knockout mice had reduced fall latency in the accelerating rotarod test, which is commonly used to evaluate motor learning and coordination in rodents [108–111]. In addition to mild motor impairment, *Grin3b* knockout mice also exhibit reduced basal activity in the home-cage behavioral test [26], which may be related to the strong expression of *Grin3b* in motor neurons [17]. In addition, NMDARs are thought to be closely related to emotional states [112–116], and ablation of *Grin3b* led to a decrease in social interactions in mice, which may cause effects on emotional states such as anxiety in mice. Interestingly, *Grin3b* knockout mice showed slight weight loss in old age, but did not cause abnormalities such as food intake and metabolism [26], and showed virtually no difference in growth from normal mice, which is consistent with mutations or deletions of *GRIN3B* in humans without a clinical phenotype. To more intuitively compare the changes in GluN3B expression in rodents, we have summarized all behavioral tests conducted in this study in Table 3. Although only very limited studies have reported *Grin3b* knockout mouse-related phenotypes, this still suggests that *Grin3b* plays an important role in non-motor-related functions in addition to motor-related regulatory roles.

**Table 3:** Behavioral changes with altered *Grin3b* expression in rodents.

Name of test	Results	Refs
General health	Normal fur, whiskers, posture, casual trials of righting reflex, whisker touch reflex, ear twitch reflex, and lifespan	[26]
Pathological examination	No pathological changes	[26]
Body weight	Lower body weight	[26]
Food intake	No significant difference	[26]
Water intake	Marginal reduction	[26]
Body temperature	Similar body temperature	[26]
Rotarod test	Latency to fall had been significantly reduced	[26]
Beam test	Reduction in the total distance	[26]
Home cage test	Decreased basal activity in their familiar environment	[26]
	Higher social interaction	[26]
Grip strength test	Similar performances	[26]
Wire hang	Similar performances	[26]
Radial maze test	No significant difference in learning	[26]
Contextual and cued fear conditioning test	No significant difference in response	[26]
Elevated plus-maze	Decreased time on open arms and number of entries	[26]
Social interaction test (novel environment)	Less social interaction	[26]
Open field test	Decrease in stereotypic activity	[26]
Light-dark transition	No significant difference	[26]
Porsolt forced swim	No significant difference	[26]

Phenotypes of rodents with altered GluN3B expression, including behavioral.

## Conclusions and Perspectives

Although the *GRIN3B* gene has a relatively high mutation rate in certain pathological settings (e.g., SCZ), *GRIN3B* null allele mutations can also be frequently detected in normal healthy people and show a global distribution with geographic variation. It has been reported that the null allele with allele frequencies of *GRIN3B* ranging between 1%–38% in some populations around the world, and with frequencies ranging between 24%–29% in the Western European population [67]. Furthermore, approximately 10% of the normal population is completely deficient in GluN3B [67]; however, this does not affect the normal life of these people; therefore, perhaps the effect of *GRIN3B* on the disease mentioned above may be race-dependent.

**Fig. 5:**
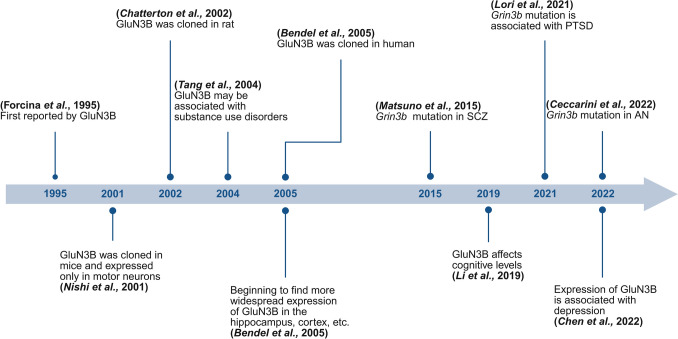
Timeline of the Discovery and Functional Studies of GluN3B. SCZ: Schizophrenia; AN: Anorexia nervosa; PTSD: Post-traumatic stress disorder. (Created in https://BioRender.com).

For mice, however, the effect of *Grin3b* deletion on their physiological function is not clear. However, with the exception of this article mentioned above [26], few other preclinical studies have attempted to explore the consequences of reduced GluN3B expression by constructing transgenic mouse lines. It has been found that *Grin3b* knockout mice would have abnormalities in immunity, body weight, and other phenotypes related to psychiatric diseases, such as hypoactivity and impairments in motor coordination, suggesting that GluN3B may have a wider function in various tissues and organs. Perhaps most importantly, in addition to CNS disorders, GluN3B could possibly be an important regulator for some non-CNS-related diseases. As shown in Fig. 5, GluN3B was first discovered in 1995, and in recent years, it has gradually been found to be potentially associated with a variety of psychiatric disorders. However, in view of the fact that the research on GluN3B is very limited, scientists need to carry out in-depth studies to explore the physiopathological roles of GluN3B in these pathological settings and fully explain the underlying mechanisms.

In summary, our study summarizes the current physiopathological studies on GluN3B, which not only deepens our understanding of its neural signaling mechanism but also provides new clues for the mechanistic studies and drug development of related neurological diseases.

## References

[1] Paoletti P, Bellone C, Zhou Q. NMDA receptor subunit diversity: impact on receptor properties, synaptic plasticity and disease. Nat Rev Neurosci 2013, 14: 383–400. 23686171 10.1038/nrn3504

[2] Pachernegg S, Strutz-Seebohm N, Hollmann M. GluN3 subunit-containing NMDA receptors: Not just one-trick ponies. Trends Neurosci 2012, 35: 240–249. 22240240 10.1016/j.tins.2011.11.010

[3] Ciabarra AM, Sullivan JM, Gahn LG, Pecht G, Heinemann S, Sevarino KA. Cloning and characterization of Chi-1: A developmentally regulated member of a novel class of the ionotropic glutamate receptor family. J Neurosci 1995, 15: 6498–6508. 7472412 10.1523/JNEUROSCI.15-10-06498.1995 PMC6577996

[4] Sucher NJ, Akbarian S, Chi CL, Leclerc CL, Awobuluyi M, Deitcher DL. Developmental and regional expression pattern of a novel NMDA receptor-like subunit (NMDAR-L) in the rodent brain. J Neurosci 1995, 15: 6509–6520. 7472413 10.1523/JNEUROSCI.15-10-06509.1995 PMC6578025

[5] Nishi M, Hinds H, Lu HP, Kawata M, Hayashi Y. Motoneuron-specific expression of NR3B, a novel NMDA-type glutamate receptor subunit that works in a dominant-negative manner. J Neurosci 2001, 21: Rc185. 11717388 10.1523/JNEUROSCI.21-23-j0003.2001 PMC6763906

[6] Andersson O, Stenqvist A, Attersand A, von Euler G. Nucleotide sequence, genomic organization, and chromosomal localization of genes encoding the human NMDA receptor subunits NR3A and NR3B. Genomics 2001, 78: 178–184. 11735224 10.1006/geno.2001.6666

[7] Stroebel D, Mony L, Paoletti P. *Glycine* agonism in ionotropic glutamate receptors. Neuropharmacology 2021, 193: 108631. 34058193 10.1016/j.neuropharm.2021.108631

[8] Traynelis SF, Wollmuth LP, McBain CJ, Menniti FS, Vance KM, Ogden KK, *et al*. Glutamate receptor ion channels: Structure, regulation, and function. Pharmacol Rev 2010, 62: 405–496. 20716669 10.1124/pr.109.002451 PMC2964903

[9] Vyklicky V, Korinek M, Smejkalova T, Balik A, Krausova B, Kaniakova M, *et al*. Structure, function, and pharmacology of NMDA receptor channels. Physiol Res 2014, 63: S191-203. 24564659 10.33549/physiolres.932678

[10] Yao Y, Harrison CB, Freddolino PL, Schulten K, Mayer ML. Molecular mechanism of ligand recognition by NR3 subtype glutamate receptors. EMBO J 2008, 27: 2158–2170. 18636091 10.1038/emboj.2008.140 PMC2516888

[11] Bendel O, Meijer B, Hurd Y, von Euler G. Cloning and expression of the human NMDA receptor subunit NR3B in the adult human hippocampus. Neurosci Lett 2005, 377: 31–36. 15722182 10.1016/j.neulet.2004.11.064

[12] Hajdú T, Juhász T, Szűcs-Somogyi C, Rácz K, Zákány R. NR1 and NR3B composed intranuclear *N*-methyl-d-aspartate receptor complexes in human melanoma cells. Int J Mol Sci 1929, 2018: 19. 10.3390/ijms19071929 PMC6073738 29966365

[13] Zhou X, Ding Q, Chen Z, Yun H, Wang H. Involvement of the GluN2A and GluN2B subunits in synaptic and extrasynaptic N-methyl-D-aspartate receptor function and neuronal excitotoxicity. J Biol Chem 2013, 288: 24151–24159. 23839940 10.1074/jbc.M113.482000 PMC3745357

[14] Pérez-Otaño I, Larsen RS, Wesseling JF. Emerging roles of GluN3-containing NMDA receptors in the CNS. Nat Rev Neurosci 2016, 17: 623–635. 27558536 10.1038/nrn.2016.92

[15] Henson MA, Roberts AC, Pérez-Otaño I, Philpot BD. Influence of the NR3A subunit on NMDA receptor functions. Prog Neurobiol 2010, 91: 23–37. 20097255 10.1016/j.pneurobio.2010.01.004 PMC2883719

[16] Kehoe LA, Bernardinelli Y, Muller D. GluN3A: An NMDA receptor subunit with exquisite properties and functions. Neural Plast 2013, 2013: 145387. 24386575 10.1155/2013/145387 PMC3872238

[17] Cavara NA, Hollmann M. Shuffling the deck anew: How NR3 tweaks NMDA receptor function. Mol Neurobiol 2008, 38: 16–26. 18654865 10.1007/s12035-008-8029-9

[18] David Sweatt J. Neural plasticity and behavior - sixty years of conceptual advances. J Neurochem 2016, 139(Suppl 2): 179–199. 26875778 10.1111/jnc.13580

[19] Crawley O, Conde-Dusman MJ, Pérez-Otaño I. GluN3A NMDA receptor subunits: More enigmatic than ever? J Physiol 2022, 600: 261–276. 33942912 10.1113/JP280879

[20] Fukaya M, Hayashi Y, Watanabe M. NR2 to NR3B subunit switchover of NMDA receptors in early postnatal motoneurons. Eur J Neurosci 2005, 21: 1432–1436. 15813953 10.1111/j.1460-9568.2005.03957.x

[21] Ishihama K, Turman JE Jr. NR3 protein expression in trigeminal neurons during postnatal development. Brain Res 2006, 1095: 12–16. 16709403 10.1016/j.brainres.2006.04.010

[22] Wee KS, Tan FCK, Cheong YP, Khanna S, Low CM. Ontogenic profile and synaptic distribution of GluN3 proteins in the rat brain and hippocampal neurons. Neurochem Res 2016, 41: 290–297. 26700428 10.1007/s11064-015-1794-8

[23] Wong HK, Liu XB, Matos MF, Chan SF, Pérez-Otaño I, Boysen M, *et al*. Temporal and regional expression of NMDA receptor subunit NR3A in the mammalian brain. J Comp Neurol 2002, 450: 303–317. 12209845 10.1002/cne.10314

[24] Matsuda K, Kamiya Y, Matsuda S, Yuzaki M. Cloning and characterization of a novel NMDA receptor subunit NR3B: A dominant subunit that reduces calcium permeability. Brain Res Mol Brain Res 2002, 100: 43–52. 12008020 10.1016/s0169-328x(02)00173-0

[25] Wee KS, Zhang Y, Khanna S, Low CM. Immunolocalization of NMDA receptor subunit NR3B in selected structures in the rat forebrain, cerebellum, and lumbar spinal cord. J Comp Neurol 2008, 509: 118–135. 18425811 10.1002/cne.21747

[26] Niemann S, Kanki H, Fukui Y, Takao K, Fukaya M, Hynynen MN, *et al*. Genetic ablation of NMDA receptor subunit NR3B in mouse reveals motoneuronal and nonmotoneuronal phenotypes. Eur J Neurosci 2007, 26: 1407–1420. 17880385 10.1111/j.1460-9568.2007.05774.x

[27] Matsuda K, Fletcher M, Kamiya Y, Yuzaki M. Specific assembly with the NMDA receptor 3B subunit controls surface expression and calcium permeability of NMDA receptors. J Neurosci 2003, 23: 10064–10073. 14602821 10.1523/JNEUROSCI.23-31-10064.2003 PMC6740865

[28] Gardoni F, Di Luca M. Protein-protein interactions at the NMDA receptor complex: From synaptic retention to synaptonuclear protein messengers. Neuropharmacology 2021, 190: 108551. 33819458 10.1016/j.neuropharm.2021.108551

[29] Dore K, Carrico Z, Alfonso S, Marino M, Koymans K, Kessels HW, *et al*. PSD-95 protects synapses from β-amyloid. Cell Rep 2021, 35: 109194. 34077732 10.1016/j.celrep.2021.109194 PMC8237704

[30] Berg LK, Larsson M, Morland C, Gundersen V. Pre- and postsynaptic localization of NMDA receptor subunits at hippocampal mossy fibre synapses. Neuroscience 2013, 230: 139–150. 23159309 10.1016/j.neuroscience.2012.10.061

[31] Perez-Otano I, Schulteis CT, Contractor A, Lipton SA, Trimmer JS, Sucher NJ, *et al*. Assembly with the NR1 subunit is required for surface expression of NR3A-containing NMDA receptors. J Neurosci 2001, 21: 1228–1237. 11160393 10.1523/JNEUROSCI.21-04-01228.2001 PMC6762235

[32] Bowery NG, Smart TG. GABA and *Glycine* as neurotransmitters: A brief history. Br J Pharmacol 2006, 147: S109-119. 16402094 10.1038/sj.bjp.0706443 PMC1760744

[33] Legendre P. The glycinergic inhibitory synapse. Cell Mol Life Sci 2001, 58: 760–793. 11437237 10.1007/PL00000899 PMC11337367

[34] Piña-Crespo JC, Talantova M, Micu I, States B, Chen HS, Tu S, *et al*. Excitatory *Glycine* responses of CNS myelin mediated by NR1/NR3 “NMDA” receptor subunits. J Neurosci 2010, 30: 11501–11505. 20739572 10.1523/JNEUROSCI.1593-10.2010 PMC2941801

[35] Chen PE, Geballe MT, Katz E, Erreger K, Livesey MR, O’Toole KK, *et al*. Modulation of *G**lycine* potency in rat recombinant NMDA receptors containing chimeric NR2A/2D subunits expressed in Xenopus *laevisoocytes*. J Physiol 2008, 586: 227–245. 17962328 10.1113/jphysiol.2007.143172 PMC2375544

[36] Grand T, Abi Gerges S, David M, Diana MA, Paoletti P. Unmasking GluN1/GluN3A excitatory *Glycine* NMDA receptors. Nat Commun 2018, 9: 4769. 30425244 10.1038/s41467-018-07236-4 PMC6233196

[37] Chatterton JE, Awobuluyi M, Premkumar LS, Takahashi H, Talantova M, Shin Y, *et al*. Excitatory *Glycine* receptors containing the NR3 family of NMDA receptor subunits. Nature 2002, 415: 793–798. 11823786 10.1038/nature715

[38] Thetford Smothers C, Woodward JJ. Pharmacological characterization of *Glycine*-activated currents in HEK 293 cells expressing N-methyl-D-aspartate NR1 and NR3 subunits. J Pharmacol Exp Ther 2007, 322: 739–748. 17502428 10.1124/jpet.107.123836

[39] Berger AJ, Dieudonné S, Ascher P. Glycine uptake governs *Glycine* site occupancy at NMDA receptors of excitatory synapses. J Neurophysiol 1998, 80: 3336–3340. 9862928 10.1152/jn.1998.80.6.3336

[40] Madry C, Mesic I, Bartholomäus I, Nicke A, Betz H, Laube B. Principal role of NR3 subunits in NR1/NR3 excitatory Glycine receptor function. Biochem Biophys Res Commun 2007, 354: 102–108. 17214961 10.1016/j.bbrc.2006.12.153

[41] Kvist T, Greenwood JR, Hansen KB, Traynelis SF, Bräuner-Osborne H. Structure-based discovery of antagonists for GluN3-containing N-methyl-D-aspartate receptors. Neuropharmacology 2013, 75: 324–336. 23973313 10.1016/j.neuropharm.2013.08.003 PMC3865070

[42] Hemelikova K, Kolcheva M, Skrenkova K, Kaniakova M, Horak M. Lectins modulate the functional properties of GluN1/GluN3-containing NMDA receptors. Neuropharmacology 2019, 157: 107671. 31202607 10.1016/j.neuropharm.2019.107671

[43] Hansen KB, Ogden KK, Yuan H, Traynelis SF. Distinct functional and pharmacological properties of Triheteromeric GluN1/GluN2A/GluN2B NMDA receptors. Neuron 2014, 81: 1084–1096. 24607230 10.1016/j.neuron.2014.01.035 PMC3957490

[44] Stroebel D, Carvalho S, Grand T, Zhu S, Paoletti P. Controlling NMDA receptor subunit composition using ectopic retention signals. J Neurosci 2014, 34: 16630–16636. 25505316 10.1523/JNEUROSCI.2736-14.2014 PMC6608501

[45] McClymont DW, Harris J, Mellor IR. Open-channel blockade is less effective on GluN3B than GluN3A subunit-containing NMDA receptors. Eur J Pharmacol 2012, 686: 22–31. 22564863 10.1016/j.ejphar.2012.04.036 PMC3657159

[46] Huang X, Sun X, Wang Q, Zhang J, Wen H, Chen WJ, *et al*. Structural insights into the diverse actions of magnesium on NMDA receptors. Neuron 2025, 113: 1006-1018.e4. 40010346 10.1016/j.neuron.2025.01.021

[47] Burnashev N, Schoepfer R, Monyer H, Ruppersberg JP, Günther W, Seeburg PH, *et al*. Control by asparagine residues of calcium permeability and magnesium blockade in the NMDA receptor. Science 1992, 257: 1415–1419. 1382314 10.1126/science.1382314

[48] Kashiwagi K, Masuko T, Nguyen CD, Kuno T, Tanaka I, Igarashi K, *et al*. Channel blockers acting at N-methyl-D-aspartate receptors: Differential effects of mutations in the vestibule and ion channel pore. Mol Pharmacol 2002, 61: 533–545. 11854433 10.1124/mol.61.3.533

[49] Cavara NA, Orth A, Hicking G, Seebohm G, Hollmann M. Residues at the tip of the pore loop of NR3B-containing NMDA receptors determine Ca^2+^ permeability and Mg^2+^ block. BMC Neurosci 2010, 11: 133. 20958962 10.1186/1471-2202-11-133 PMC2974739

[50] Zhu Z, Yi F, Epplin MP, Liu D, Summer SL, Mizu R, *et al*. Negative allosteric modulation of GluN1/GluN3 NMDA receptors. Neuropharmacology 2020, 176: 108117. 32389749 10.1016/j.neuropharm.2020.108117 PMC7530031

[51] Xiong K, Lou S, Lian Z, Wu Y, Kou Z. The GluN3-containing NMDA receptors. Channels (Austin) 2025, 19: 2490308. 40235311 10.1080/19336950.2025.2490308 PMC12005412

[52] Skrenkova K, Hemelikova K, Kolcheva M, Kortus S, Kaniakova M, Krausova B, *et al*. Structural features in the *Glycine*-binding sites of the GluN1 and GluN3A subunits regulate the surface delivery of NMDA receptors. Sci Rep 2019, 9: 12303. 31444392 10.1038/s41598-019-48845-3 PMC6707325

[53] Zeng Y, Zheng Y, Zhang T, Ye F, Zhan L, Kou Z, *et al*. Identification of a subtype-selective allosteric inhibitor of GluN1/GluN3 NMDA receptors. Front Pharmacol 2022, 13: 888308. 35754487 10.3389/fphar.2022.888308 PMC9218946

[54] Soukup O, Jun D, Zdarova-Karasova J, Patocka J, Musilek K, Korabecny J, *et al*. A resurrection of 7-MEOTA: A comparison with tacrine. Curr Alzheimer Res 2013, 10: 893–906. 24093535 10.2174/1567205011310080011

[55] Kaniakova M, Kleteckova L, Lichnerova K, Holubova K, Skrenkova K, Korinek M, *et al*. 7-Methoxyderivative of tacrine is a ‘foot-in-the-door’ open-channel blocker of GluN1/GluN2 and GluN1/GluN3 NMDA receptors with neuroprotective activity in vivo. Neuropharmacology 2018, 140: 217–232. 30099049 10.1016/j.neuropharm.2018.08.010

[56] Lee J, Green MF. Social preference and glutamatergic dysfunction: Underappreciated prerequisites for social dysfunction in schizophrenia. Trends Neurosci 2016, 39: 587–596. 27477199 10.1016/j.tins.2016.06.005 PMC5951176

[57] Amoah SK, Rodriguez BA, Logothetis CN, Chander P, Sellgren CM, Weick JP, *et al*. Exosomal secretion of a psychosis-altered miRNA that regulates glutamate receptor expression is affected by antipsychotics. Neuropsychopharmacology 2020, 45: 656–665. 31775160 10.1038/s41386-019-0579-1 PMC7021900

[58] Mahmoud GS, Hosny G, Sayed SA. The protective effect of olanzapine on ketamine induced cognitive deficit and increased NR1 expression in rat model of schizophrenia. Int J Physiol Pathophysiol Pharmacol 2021, 13: 22–35. 34093963 PMC8166812

[59] Krzystanek M, Pałasz A. NMDA receptor model of antipsychotic drug-induced hypofrontality. Int J Mol Sci 2019, 20: 1442. 30901926 10.3390/ijms20061442 PMC6471005

[60] Krystal JH, Karper LP, Seibyl JP, Freeman GK, Delaney R, Bremner JD, *et al*. Subanesthetic effects of the noncompetitive NMDA antagonist, ketamine, in humans. Psychotomimetic, perceptual, cognitive, and neuroendocrine responses. Arch Gen Psychiatry 1994, 51: 199–214. 8122957 10.1001/archpsyc.1994.03950030035004

[61] Alsabban AH, Morikawa M, Tanaka Y, Takei Y, Hirokawa N. Kinesin Kif3b mutation reduces NMDAR subunit NR2A trafficking and causes schizophrenia-like phenotypes in mice. EMBO J 2020, 39: e101090. 31746486 10.15252/embj.2018101090 PMC6939202

[62] Mohn AR, Gainetdinov RR, Caron MG, Koller BH. Mice with reduced NMDA receptor expression display behaviors related to schizophrenia. Cell 1999, 98: 427–436. 10481908 10.1016/s0092-8674(00)81972-8

[63] Burket JA, Deutsch SI. Metabotropic functions of the NMDA receptor and an evolving rationale for exploring NR2A-selective positive allosteric modulators for the treatment of autism spectrum disorder. Prog Neuropsychopharmacol Biol Psychiatry 2019, 90: 142–160. 30481555 10.1016/j.pnpbp.2018.11.017

[64] Myers SJ, Yuan H, Kang JQ, Tan FCK, Traynelis SF, Low CM. Distinct roles of GRIN2A and GRIN2B variants in neurological conditions. F1000Res 2019, 8: F1000FacultyRev-F1000Faculty1940. 10.12688/f1000research.18949.1 PMC6871362 31807283

[65] Yu Y, Lin Y, Takasaki Y, Wang C, Kimura H, Xing J, *et al*. Rare loss of function mutations in N-methyl-D-aspartate glutamate receptors and their contributions to schizophrenia susceptibility. Transl Psychiatry 2018, 8: 12. 29317596 10.1038/s41398-017-0061-y PMC5802496

[66] Tarabeux J, Kebir O, Gauthier J, Hamdan FF, Xiong L, Piton A, *et al*. Rare mutations in N-methyl-D-aspartate glutamate receptors in autism spectrum disorders and schizophrenia. Transl Psychiatry 2011, 1: e55. 22833210 10.1038/tp.2011.52 PMC3309470

[67] Niemann S, Landers JE, Churchill MJ, Hosler B, Sapp P, Speed WC, *et al*. Motoneuron-specific NR3B gene: No association with ALS and evidence for a common null allele. Neurology 2008, 70: 666–676. 17687115 10.1212/01.wnl.0000271078.51280.17

[68] Putnam DK, Sun J, Zhao Z. Exploring schizophrenia drug-gene interactions through molecular network and pathway modeling. AMIA Annu Symp Proc 2011, 2011: 1127–1133. 22195173 PMC3243134

[69] Hirschfeldova K, Cerny J, Bozikova P, Kuchtiak V, Rausch T, Benes V, *et al*. Evidence for the association between the intronic haplotypes of ionotropic glutamate receptors and first-episode schizophrenia. J Pers Med 2021, 11: 1250. 34945722 10.3390/jpm11121250 PMC8708351

[70] Hornig T, Grüning B, Kundu K, Houwaart T, Backofen R, Biber K, *et al*. GRIN3B missense mutation as an inherited risk factor for schizophrenia: Whole-exome sequencing in a family with a familiar history of psychotic disorders. Genet Res (Camb) 2017, 99: e1. 28132660 10.1017/S0016672316000148 PMC6865172

[71] Matsuno H, Ohi K, Hashimoto R, Yamamori H, Yasuda Y, Fujimoto M, *et al*. A naturally occurring null variant of the NMDA type glutamate receptor NR3B subunit is a risk factor of schizophrenia. PLoS One 2015, 10: e0116319. 25768306 10.1371/journal.pone.0116319 PMC4358936

[72] Lin YT, Hsieh MH, Liu CC, Hwang TJ, Chien YL, Hwu HG, *et al*. A recently-discovered NMDA receptor gene, GRIN3B, is associated with duration mismatch negativity. Psychiatry Res 2014, 218: 356–358. 24814139 10.1016/j.psychres.2014.04.032

[73] Volkow ND, Blanco C. Substance use disorders: A comprehensive update of classification, epidemiology, neurobiology, clinical aspects, treatment and prevention. World Psychiatry 2023, 22: 203–229. 37159360 10.1002/wps.21073 PMC10168177

[74] Tang W, Wesley M, Freeman WM, Liang B, Hemby SE. Alterations in ionotropic glutamate receptor subunits during binge cocaine self-administration and withdrawal in rats. J Neurochem 2004, 89: 1021–1033. 15140200 10.1111/j.1471-4159.2004.02392.x PMC3843358

[75] Sedaghati M, Vousooghi N, Goodarzi A, Yaghmaei P, Mokri A, Zarrindast MR. Expression of NR3B but not NR2D subunit of NMDA receptor in human blood lymphocytes can serve as a suitable peripheral marker for opioid addiction studies. Eur J Pharmacol 2010, 633: 50–54. 20153313 10.1016/j.ejphar.2010.02.007

[76] Xie X, Liu H, Zhang J, Chen W, Zhuang D, Duan S, *et al*. Association between genetic variations of NMDA receptor NR3 subfamily genes and heroin addiction in male Han Chinese. Neurosci Lett 2016, 631: 122–125. 27542340 10.1016/j.neulet.2016.08.025

[77] Liu HP, Lin WY, Liu SH, Wang WF, Tsai CH, Wu BT, *et al*. Genetic variation in N-methyl-D-aspartate receptor subunit NR3A but not NR3B influences susceptibility to Alzheimer’s disease. Dement Geriatr Cogn Disord 2009, 28: 521–527. 20016182 10.1159/000254757

[78] Sadat-Shirazi MS, Vousooghi N, Alizadeh B, Makki SM, Zarei SZ, Nazari S, *et al*. Expression of NMDA receptor subunits in human blood lymphocytes: A peripheral biomarker in online computer game addiction. J Behav Addict 2018, 7: 260–268. 29788757 10.1556/2006.7.2018.35 PMC6174581

[79] Nestler EJ, Lüscher C. The molecular basis of drug addiction: Linking epigenetic to synaptic and circuit mechanisms. Neuron 2019, 102: 48–59. 30946825 10.1016/j.neuron.2019.01.016 PMC6587180

[80] Lüscher C, Malenka RC. Drug-evoked synaptic plasticity in addiction: From molecular changes to circuit remodeling. Neuron 2011, 69: 650–663. 21338877 10.1016/j.neuron.2011.01.017 PMC4046255

[81] Yuan T, Mameli M, O’Connor EC, Dey PN, Verpelli C, Sala C, *et al*. Expression of cocaine-evoked synaptic plasticity by GluN3A-containing NMDA receptors. Neuron 2013, 80: 1025–1038. 24183704 10.1016/j.neuron.2013.07.050

[82] Chen J, Luo Y, Liang X, Kong X, Xiao Q, Tang J, *et al*. Alteration in NMDAR subunits in different brain regions of chronic unpredictable mild stress (CUMS) rat model. Transl Neurosci 2022, 13: 379–389. 36348956 10.1515/tnsci-2022-0255 PMC9601380

[83] Martín-Sánchez A, Piñero J, Nonell L, Arnal M, Ribe EM, Nevado-Holgado A, *et al*. Comorbidity between Alzheimer’s disease and major depression: A behavioural and transcriptomic characterization study in mice. Alzheimers Res Ther 2021, 13: 73. 33795014 10.1186/s13195-021-00810-x PMC8017643

[84] Lee LC, Su MT, Huang HY, Cho YC, Yeh TK, Chang CY. Association of CaMK2A and MeCP2 signaling pathways with cognitive ability in adolescents. Mol Brain 2021, 14: 152. 34607601 10.1186/s13041-021-00858-8 PMC8491411

[85] Li XY, Hu P, Li QY, Zhang M, Lai QW, Wang X, *et al*. Correlations between the level of antibody against peptide of glutamate receptor NR3B subunit in the CSF and cognitive comorbidities of patients with epilepsy. Eur Rev Med Pharmacol Sci 2019, 23: 328–337. 30657574 10.26355/eurrev_201901_16780

[86] Wen Y, Fu Z, Li J, Liu M, Wang X, Chen J, *et al*. Targeting m^6^A mRNA demethylase FTO alleviates manganese-induced cognitive memory deficits in mice. J Hazard Mater 2024, 476: 134969. 38908185 10.1016/j.jhazmat.2024.134969

[87] Ceccarini MR, Precone V, Manara E, Paolacci S, Maltese PE, Benfatti V, *et al*. A next generation sequencing gene panel for use in the diagnosis of anorexia nervosa. Eat Weight Disord 2022, 27: 1869–1880. 34822136 10.1007/s40519-021-01331-0

[88] Donato K, Medori MC, Macchia A, Cecchin S, Ceccarini MR, Beccari T, *et al*. Genetic variants identified in novel candidate genes for anorexia nervosa and analysis of molecular pathways for diagnostic applications. Eur Rev Med Pharmacol Sci 2023, 27: 77–88. 38112957 10.26355/eurrev_202312_34692

[89] Lori A, Schultebraucks K, Galatzer-Levy I, Daskalakis NP, Katrinli S, Smith AK, *et al*. Transcriptome-wide association study of post-trauma symptom trajectories identified GRIN3B as a potential biomarker for PTSD development. Neuropsychopharmacology 2021, 46: 1811–1820. 34188182 10.1038/s41386-021-01073-8 PMC8357796

[90] Albrechet-Souza L, Gilpin NW. The predator odor avoidance model of post-traumatic stress disorder in rats. Behav Pharmacol 2019, 30: 105–114. 30640179 10.1097/FBP.0000000000000460 PMC6422743

[91] Endres T, Fendt M. Aversion- vs fear-inducing properties of 2,4,5-trimethyl-3-thiazoline, a component of fox odor, in comparison with those of butyric acid. J Exp Biol 2009, 212: 2324–2327. 19617424 10.1242/jeb.028498

[92] Deslauriers J, Toth M, Der-Avakian A, Risbrough VB. Current status of animal models of posttraumatic stress disorder: Behavioral and biological phenotypes, and future challenges in improving translation. Biol Psychiatry 2018, 83: 895–907. 29338843 10.1016/j.biopsych.2017.11.019 PMC6085893

[93] Dielenberg RA, McGregor IS. Defensive behavior in rats towards predatory odors: A review. Neurosci Biobehav Rev 2001, 25: 597–609. 11801285 10.1016/s0149-7634(01)00044-6

[94] Tyler RE, Bluitt MN, Engers JL, Lindsley CW, Besheer J. The effects of predator odor (TMT) exposure and mGlu_3_ NAM pretreatment on behavioral and NMDA receptor adaptations in the brain. Neuropharmacology 2022, 207: 108943. 35007623 10.1016/j.neuropharm.2022.108943 PMC8844221

[95] Joffe ME, Santiago CI, Engers JL, Lindsley CW, Conn PJ. Metabotropic glutamate receptor subtype 3 gates acute stress-induced dysregulation of amygdalo-cortical function. Mol Psychiatry 2019, 24: 916–927. 29269844 10.1038/s41380-017-0015-z PMC6013320

[96] Walker AG, Wenthur CJ, Xiang Z, Rook JM, Emmitte KA, Niswender CM, *et al*. Metabotropic glutamate receptor 3 activation is required for long-term depression in medial prefrontal cortex and fear extinction. Proc Natl Acad Sci U S A 2015, 112: 1196–1201. 25583490 10.1073/pnas.1416196112 PMC4313856

[97] Christianson JP, Jennings JH, Ragole T, Flyer JGN, Benison AM, Barth DS, *et al*. Safety signals mitigate the consequences of uncontrollable stress via a circuit involving the sensory insular cortex and bed nucleus of the stria *Terminalis*. Biol Psychiatry 2011, 70: 458–464. 21684526 10.1016/j.biopsych.2011.04.004 PMC3159417

[98] Alvarez RP, Kirlic N, Misaki M, Bodurka J, Rhudy JL, Paulus MP, *et al*. Increased anterior *Insula* activity in anxious individuals is linked to diminished perceived control. Transl Psychiatry 2015, 5: e591. 26125154 10.1038/tp.2015.84 PMC4490294

[99] Avazzadeh S, Quinlan LR, Reilly J, McDonagh K, Jalali A, Wang Y, *et al*. NRXN1α^+/-^ is associated with increased excitability in ASD iPSC-derived neurons. BMC Neurosci 2021, 22: 56. 34525970 10.1186/s12868-021-00661-0 PMC8442436

[100] Chen K, Kardys A, Chen Y, Flink S, Tabakoff B, Shih JC. Altered gene expression in early postnatal monoamine oxidase A knockout mice. Brain Res 2017, 1669: 18–26. 28535982 10.1016/j.brainres.2017.05.017 PMC5531263

[101] Tameh AA, Karimian M, Zare-Dehghanani Z, Aftabi Y, Beyer C. Role of steroid therapy after ischemic stroke by n-methyl-d-aspartate receptor gene regulation. J Stroke Cerebrovasc Dis 2018, 27: 3066–3075. 30072177 10.1016/j.jstrokecerebrovasdis.2018.06.041

[102] Chen J, Zhang J, Yang DD, Li ZC, Zhao B, Chen Y, *et al*. Clonidine ameliorates cerebral ischemia-reperfusion injury by up-regulating the GluN3 subunits of NMDA receptor. Metab Brain Dis 2022, 37: 1829–1841. 35727521 10.1007/s11011-022-01028-y

[103] Camafort M, Kreutz R, Cho MC. Diagnosis and management of resistant hypertension. Heart 2024, 110: 1336–1342. 38135468 10.1136/heartjnl-2022-321730

[104] Jabir NR, Firoz CK, Zughaibi TA, Alsaadi MA, Abuzenadah AM, Al-Asmari AI, *et al*. A literature perspective on the pharmacological applications of yohimbine. Ann Med 2022, 54: 2861–2875. 36263866 10.1080/07853890.2022.2131330 PMC9590431

[105] Micu I, Jiang Q, Coderre E, Ridsdale A, Zhang L, Woulfe J, *et al*. NMDA receptors mediate calcium accumulation in myelin during chemical ischaemia. Nature 2006, 439: 988–992. 16372019 10.1038/nature04474

[106] Káradóttir R, Cavelier P, Bergersen LH, Attwell D. NMDA receptors are expressed in oligodendrocytes and activated in ischaemia. Nature 2005, 438: 1162–1166. 16372011 10.1038/nature04302 PMC1416283

[107] Stys PK, Lipton SA. White matter NMDA receptors: An unexpected new therapeutic target? Trends Pharmacol Sci 2007, 28: 561–566. 17961731 10.1016/j.tips.2007.10.003

[108] Smeets CJLM, Jezierska J, Watanabe H, Duarri A, Fokkens MR, Meijer M, *et al*. Elevated mutant dynorphin A causes Purkinje cell loss and motor dysfunction in spinocerebellar *Ataxia* type 23. Brain 2015, 138: 2537–2552. 26169942 10.1093/brain/awv195

[109] Achilly NP, Wang W, Zoghbi HY. Presymptomatic training mitigates functional deficits in a mouse model of Rett syndrome. Nature 2021, 592: 596–600. 33762729 10.1038/s41586-021-03369-7 PMC8093094

[110] Esposito MS, Capelli P, Arber S. Brainstem nucleus MdV mediates skilled forelimb motor tasks. Nature 2014, 508: 351–356. 24487621 10.1038/nature13023

[111] Bieri G, Brahic M, Bousset L, Couthouis J, Kramer NJ, Ma R, *et al*. LRRK2 modifies α-syn pathology and spread in mouse models and human neurons. Acta Neuropathol 2019, 137: 961–980. 30927072 10.1007/s00401-019-01995-0 PMC6531417

[112] Ma S, Chen M, Jiang Y, Xiang X, Wang S, Wu Z, *et al*. Sustained antidepressant effect of ketamine through NMDAR trapping in the LHb. Nature 2023, 622: 802–809. 37853123 10.1038/s41586-023-06624-1 PMC10600008

[113] Masella G, Silva F, Corti E, Azkona G, Madeira MF, Tomé ÂR, *et al*. The amygdala NT3-TrkC pathway underlies inter-individual differences in fear extinction and related synaptic plasticity. Mol Psychiatry 2024, 29: 1322–1337. 38233468 10.1038/s41380-024-02412-z PMC11189811

[114] Taylor Flynn L, Gao WJ. DNA methylation and the opposing NMDAR dysfunction in schizophrenia and major depression disorders: A converging model for the therapeutic effects of psychedelic compounds in the treatment of psychiatric illness. Mol Psychiatry 2023, 28: 4553–4567. 37679470 10.1038/s41380-023-02235-4 PMC11034997

[115] Lee B, Pothula S, Wu M, Kang H, Girgenti MJ, Picciotto MR, *et al*. Positive modulation of N-methyl-D-aspartate receptors in the mPFC reduces the spontaneous recovery of fear. Mol Psychiatry 2022, 27: 2580–2589. 35418600 10.1038/s41380-022-01498-7 PMC9135632

[116] Hua SS, Ding JJ, Sun TC, Guo C, Zhang Y, Yu ZH, *et al*. NMDA receptor-dependent synaptic potentiation via APPL1 signaling is required for the accessibility of a prefrontal neuronal assembly in retrieving fear extinction. Biol Psychiatry 2023, 94: 262–277. 36842495 10.1016/j.biopsych.2023.02.013

